# Emotion dysregulation acts in the relationship between vulnerable narcissism and suicidal ideation

**DOI:** 10.1111/sjop.12730

**Published:** 2021-05-06

**Authors:** Sara Ponzoni, Sara Beomonte Zobel, Guyonne Rogier, Patrizia Velotti

**Affiliations:** ^1^ Department of Educational Sciences University of Genoa Italy; ^2^ Department of Dynamic and Clinical Psychology Faculty of Medicine and Psychology Sapienza University of Rome Italy

**Keywords:** Emotion dysregulation, grandiose narcissism, pathological narcissism, suicide ideation, vulnerable narcissism

## Abstract

Suicide behaviors are peculiar aspects of several cluster B disorders, including Narcissistic Personality Disorder. To date, it is still unclear which facet of narcissism is more related to the desire to die and which other factors are involved in this relationship. This study aims to offer preliminary empirical evidences concerning the relationship between narcissism, emotion dysregulation and suicide ideation. We administered the Pathological Narcissism Inventory (PNI), Difficulties in Emotion Regulation Scale (DERS), PID‐5‐BF (Personality Inventory for DSM‐5‐Short Form) and Beck Scale for Suicide ideation (BSI) to a sample of individuals with suicide ideation (*n* = 70) and a sample of community participants (*n* = 154). Controlling for age, gender and Negative Affectivity, we found that BSI scores correlated significantly with the vulnerable dimension of narcissism, but not with the grandiose one, and with all DERS dimension, apart from Awareness. Nevertheless, emotion dysregulation moderates the relationship between vulnerable narcissism and suicide ideation. Suicide ideation seems to be deeply connected with the vulnerable dimension of pathological narcissism and the relationship between the constructs is totally mediated by emotion dysregulation. Future directions and clinical implications are discussed.

## Introduction

Suicide ideation is defined as a desire to engage in suicide and/or to plan suicide attempts (O’Carroll, Berman, Maris, Moscicki, Tanney & Silverman, 1996). Research concerning risk factors for suicide ideation has implicated diverse features (Klonsky, May & Saffer, 2016), including personality disorders (May, Klonsky & Klein, 2012) and emotion dysregulation (Ong & Thompson, 2018). The majority of the studies examining the relationship between suicide behavior (i.e., any action that could cause a person to die) and personality disorders have been conducted among individuals with borderline personality disorders. However, other personality disorders were positively linked to suicidal behaviors, including suicide ideation (Szücs, Szanto, Aubry, & Dombrovski, 2018).

### A look at narcissism

Narcissism is considered the basis for validation and self‐affirmation needs (Pincus, Ansell, Pimentel, Cain, Wright & Levy, 2009), when it results in pathological outcomes the adaptive mechanisms to maintain a relatively positive image of itself are compromised (Kealy & Rasmussen, 2011). In these cases, difficulties are observed in self‐esteem and interpersonal relationships, but also in the elaboration of useful strategies to face disappointments, failures and threats to one’s image. Struggles in managing such intense validation and admiration needs lead to psychopathological consequences that manifest themselves as a grandiose essence – a type of narcissism identified by the DSM‐5 (American Psychiatric Association, 2013). Therefore, from a psychoanalytic perspective narcissistic personality disorder can be described as a continuum which sees, at one end, an envious and greedy individual who constantly requires attention from others and, on the other, a vulnerable individual sensitive to external judgments, who easily tends to self‐fragmentation (Gabbard, 2014).

The grandiose manifestation of narcissism appears to be characterized by dominance, revenge, interpersonal exploitation, experimentation of anger and aggression, constant search for self‐improvement and lack of awareness of the impact that his behaviors may have on others (Dickinson & Pincus, 2003). Similarly, to the grandiose type, even in vulnerable narcissism when grandiose expectations are despised, outbursts of anger and hostility are generated, but in this case these reactions are followed by experiences of shame and depression. However, fantasies and claims of grandiosity are hidden behind shyness, attention for others, fear of disapproval and others' judgment, social avoidance, emotional coldness and fear of experiencing shame because of one's needs (Dickinson & Pincus, 2003).

Assuming that both forms of narcissism present shared deficits, especially concerning identity issues, related to conflicting feelings of grandiosity and vulnerability (Given‐Wilson, McIlwain & Warburton, 2011), the substantial difference between the two types of narcissism lies in the manifest or hidden character of grandiose fantasies, although most individuals show some degree of expressive fluctuation between grandiosity and vulnerability. So, phenotypic manifestations of grandiosity and vulnerability are simply prevailing ways of managing, dealing with and manifesting the core of pathological narcissism (i.e., self‐directed preoccupation and absorption, contempt for others). Narcissistic grandiosity is characterized by an excessively positive self‐image associated with behaviors and attitudes based on characteristics of arrogance, conceit and dominance; narcissistic vulnerability is characterized by a fluctuant sense of self‐worth associated with experiences of shame, emptiness, hopelessness and social withdrawal (Akhtar, 2003; Dickinson & Pincus, 2003; Pincus *et al*., 2009; Ronningstam, 2005). Despite this, the authors stress that these characteristics must not be confused with the defining elements of the pathology, as they represent exclusively descriptive elements of the two expressive modes of pathological narcissism (Pincus & Roche, 2011).

The result of the operationalization process of this conceptualization of narcissism was the Pathological Narcissism Inventory (PNI; Pincus *et al*., 2009). Preceded by a careful review of the theoretical and empirical literature on narcissism (Cain, Pincus & Ansell, 2008), PNI was built with the explicit purpose of measuring pathological narcissism both in its grandiose and vulnerable manifestations, with particular attention not only to the behavioral aspects but also to the characteristics of the typical functioning of this pathology, that lies in the coexistence of rigid and pervasive narcissistic needs along with self‐regulation deficits. Currently, it is the only tool that allows a multidimensional evaluation of pathological narcissism (Pincus, 2013; Pincus *et al*., 2009; Wright *et al*., 2010).

### Suicide in narcissistic personalities

Personality disorders are considered an important risk factor, characterizing about 15% of suicides in hospitalized patients and almost 12% in outpatients (Bertolote & Fleishmann, 2002; Bertolote, Fleischmann, De Leo & Wasserman, 2004), especially considering cluster B disorders: Borderline Personality Disorder has the highest suicide rates, with percentages ranging from 3 to 9% depending on the samples (Bertolote *et al*., 2004; Lieb, Zanarini, Schmahl, Linehan & Bohus, 2004; Pompili, Girardi, Ruberto & Tatarelli, 2005), but Narcissistic Personality Disorder (NPD) follows immediately with percentages that are around 5%, just for completed suicides (Ansell, Wright, Markowitz *et al*., 2015). Nonetheless, the relationship between NPD and suicide has been relatively poorly investigated (Coleman, Lawrence Parekh *et al*., 2017). NPD is associated with a greater risk of death by suicide and a greater lethality of suicide attempts (compared to other personality disorders; Giner, Blasco‐Fontecilla, Perez‐Rodriguez *et al*., 2013) but it is also weakly associated with non‐fatal suicide attempts (Coleman *et al*., 2017). In addition, pathological narcissism and NPD are considered risk factors for suicide ideation among young people, adults and elderly (Pincus, Roche & Good, 2015; Ronningstam, 2011).

Overall, suicidal behaviors in individuals suffering from pathological narcissism consist in a specific type of self‐directed anger and aggression related to injuries caused to the ego by events that threat the sense of superiority and importance of the narcissist (Ronningstam & Maltsberger, 1998). These events can generate anger, loneliness and uselessness, emotions related to shame which – in turn – gives rise to an intolerable anguish, that progressively leads the person to surrender and precipitates suicide crises (Ronningstam, Weinberg & Maltsberger, 2008). Such mechanisms are complicated by impairments in emotion regulation, along with the inability to understand the emotional meaning of the experiences and the absence of an adequate mental organization, that may work as a self‐protection: when the grandiose self is threatened, anger and shame cannot be tolerated or regulated, and the self becomes vulnerable to (self) destruction (Ronningstam & Maltsberger, 1998).

Despite the evidence, it is not possible not to consider some factors that could intervene in the relationship between narcissism and suicide ideation. For instance, a number of authors have suggested that negative affectivity (i.e., the tendency to experience a wide range of negative emotions) is strongly linked to narcissism, since the instability of the perception of one's value is fluctuating and strongly dependent on feedbacks from the outside world (Rhodenwalt, Madrian & Cheeney, 1998). Furthermore, considering that depressive symptoms and negative affectivity are considered considerable risk factors for suicide ideation and behaviors (Mackenzie et al., 2011; Rojas et al., 2015), along with the fact that depression is the most common diagnosis among young adult suicidal people (Brent et al., 2002), we hypothesize that negative affectivity may act as a confounding variable in the relationship between narcissism and suicide ideation.

### Emotion dysregulation

Considering the constituent dimensions of emotion dysregulation according to Gratz and Roemer’s (2004) model, research highlights that impairments in the regulation of negative emotions are extremely linked to maladaptive behaviors (Buckholdt, Parra, Anestis *et al*., 2015; Garofalo *et al.*, 2018) and psychopathologies (Gillespie, Garofalo & Velotti, 2018; Rogier & Velotti, 2018; Sheppes, Suri & Gross, 2015), encompassing personality disorders (Dimaggio, Popolo, Montano *et al*., 2017; Garofalo *et al.*, 2018).

According to Morf and Rhodewalt (2001), the continuous search for admiration, approval and gratification can be a precise indicator of the inability to autonomously regulate one's internal states. Taking into account the relationship between narcissism and emotion dysregulation, Besser and Priel (2010) compared grandiose narcissism and vulnerable narcissism in terms of emotional reactions in response to threats related to failure in achieving results and to interpersonal refusals, pointing out that both types of narcissism have shown associations with negative emotional reactivity related to specific threatening situations (Besser & Priel, 2010). Furthermore, research conducted by Zhang, Wanga Youa Lüa and Luo (2015) has highlighted that grandiose narcissism showed significant negative correlations with the emotion regulation dimensions of awareness and emotional clarity. In contrast, vulnerable narcissism showed significant positive correlations with general difficulties in regulating emotions and in accessing adaptive regulation strategies; in particular, several deficiencies emerged in the dimensions of acceptance of emotions, clarity and impulse control (Zhang *et al*., 2015).

Difficulties in regulating negative emotions was also positively associated with suicide ideation and attempts (Hatkevich, Penner & Sharp, 2019). Neacsiu, Fang, Rodriguez and Rosenthal (2018) evidenced that problems in emotion regulation are differentially connected to suicidal behaviors. Finally, Al‐Dajani, Uliaszek and Hamdullahpur (2019) showed that, cross‐sectionally, the relationship between emotion dysregulation and suicide ideation was significantly moderated by the belief of suicide as an escape, and longitudinally the latter moderated only the relationship between lack of clarity and suicide ideation.

Emotional dysregulation can affect the sense of omnipotence and internal control and put people with narcissistic pathology in a tight spot, that is, negative emotions such as shame can activate a depressive state, narcissistic anger and self‐injurious intention at the same time (Jaksic, Marcinko, Hanzek, Rebernjak & Ogrodniczuk, 2017). In this line, Jaksic and colleagues (2017) showed that vulnerable narcissism had the strongest associations with both shame and suicide ideation. Indeed, the perception of not having effective regulatory strategies available to manage a situation, the non‐acceptance of emotions, the scarce tolerance to anguish and the negative urgency combined can determine a sense of impotence and hopelessness which can trigger suicide ideation, such as thoughts and fantasies concerning suicidal behavior, that at least could appear as an option (Rajappa, Gallagher & Miranda, 2012).

Studies that took into consideration the role of emotion dysregulation in the suicidal phenomenon highlighted its predictive value concerning suicide ideation, while the relationship with suicidal behavior, although solid, appears to be more complex and ambiguous (Selby, Anestis, Bender & Joiner, 2009; Tamás, Kovacs, Gentzler *et al*., 2007). The question of the way in which the variables examined interact in determining suicidal risk remains open. For example, the contradictions about the significant association between grandiose narcissism and both suicide ideation (in line with the study of Ellison, Levy, Cain, Ansell and Pincus (2003) and in contrast to the research by Pincus and colleagues (2009)) and emotion dysregulation (Zhang *et al*., 2015) and the fact that vulnerable narcissism shows the strongest associations with suicide ideation (Jaksic *et al*., 2017) could suggest that some components of grandiosity, such as the exaggerated sense of superiority, grandiose fantasies, interpersonal exploitation, may be protective factors against suicidal conscious thoughts; on the other hand, they could indicate that life‐long suicide attempts may be manifestations of the oscillations between grandiose and vulnerable mental states and of emotion dysregulation typical of pathological narcissism (Jaksic *et al*., 2017; Zhang *et al*., 2015).

Considering the lack of agreement in the existing literature, although the existence of correlations between these constructs, the role of narcissistic dimensions – grandiose and vulnerable – is still open to question, as the part played by emotion dysregulation. Additionally, research about the joint effect of vulnerable narcissism and emotion dysregulation on suicide ideation is still scarce.

However, it seems primary to provide evidences on the mechanisms of interplay between the different dimensions of narcissism and emotion dysregulation, in order to clarify the potential risk factors associated with narcissistic vulnerabilities accounting for suicide ideation.

## Current study

The present study aims to investigate the way in which pathological narcissism and emotion dysregulation cooperates in suicide ideation. Specifically, we intend to investigate whether some aspects of narcissism and emotion dysregulation are associated with suicide ideation hypothesizing that emotion dysregulation mediates the relationship between vulnerable narcissism and suicide ideation. The research questions for this study were: Is narcissism a risk factor for suicide ideation? Which narcissism dimension is more related to suicide ideation? Does emotion dysregulation play a role in the relationship between narcissism and suicide?

## Method

### Participants and procedure

The research reached a sample of 224 adults. The clinical sample is composed of 70 subjects (44.1% male) recruited in two Italian mental health services with a mean age of 35.18 years (*SD* = 15.62) that have come to clinical attention following suicide ideation or suicide attempts. The comparison sample is composed of 154 subjects (41.2% male) recruited through a snowball sampling, with a mean age of 27.41 years (*SD* = 12.17).

Before the involvement of each participant in the research procedure, the research’s aims and scopes were briefly exposed and information toward privacy and anonymity were consigned. Participants have completed a written consent. In addition, participants were asked to fulfill self‐report questionnaires under the supervision of a psychologist. All procedures complied with the official directions established by the American Psychological Association and were approved by the Research Ethic Board of the University of Rome ‐ Sapienza (N. 21/2018).

### Measures

Each participant completed a research protocol consisting of the following self‐administered questionnaires; An initial questionnaire collected *demographic information* (e.g., Age, Gender).

Levels of pathological narcissism have been measured through the use of the *Pathological Narcissism Inventory – PNI* (Fossati, Feeney, Pincus, Borroni & Maffei, 2015; Pincus *et al*., 2009). The questionnaire provides two main scores indicating levels of Grandiose Narcissism and Vulnerable Narcissism through the convergence of seven subscales which identify the main components of the constructs. Specifically, Exploitative, Self‐Enhancement, Grandiose Fantasy and Entitlement subscales allow to evaluate Grandiose Narcissism; equally, Contingent Self‐esteem, Hiding the Self and Devaluing subscales allow to evaluate Vulnerable Narcissism. The dimensions are evaluated through 52 items asking the participant to indicate how much each assertion describes him, answering on a Likert‐type scale ranging from 1 (“it does not describe me at all”) to 6 (“it describes me perfectly”). In our study the tool has demonstrated good reliability with the Cronbach’s alpha coefficient for PNI Grandiosity dimension equal to 0.92 and for PNI Vulnerability dimension equal to 0.93. Subscales demonstrated good reliability as well, with Cronbach’s alpha coefficients comprised between 0.75 and 0.93.

Emotion dysregulation has been evaluated through the use of the *Difficulties in Emotion Regulation* *Scale –* *DERS* (Giromini, Velotti, de Campora, Bonalume & Cesare Zavattini, 2012; Gratz & Roemer, 2004). This self‐report questionnaire is made up of six subscales (36 items) based on the six factors that reflect the multidimensional definition of emotion regulation: (1) non‐acceptance of negative emotions (Non‐acceptance); (2) inability to conduct targeted behaviors when experiencing negative emotions (Goals); (3) difficulty controlling impulsive behaviors when experiencing negative emotions (Impulse); (4) limited access to regulating strategies that are deemed effective (Strategies); (5) lack of awareness of one's emotions (Awareness); and (6) lack of understanding of the nature of one's emotional responses (Clarity). Individual patterns of emotion regulation are measured through a five‐point Likert scale (from 1 = never to 5 = always). Both the total scale and the subscales of DERS have shown good reliability, with Cronbach's alpha values equal to or greater than 0.80 for the specific subscales (except for the awareness dimension whose index was equal to 0.67) and equal to 0.95 for the total scale.

Levels of suicide ideation have been measured by the use of the *Beck* *Scale for Suicide ideation – BSI* (Beck, Brown & Steer, 1997; Beck, Kovacs & Weissman, 1979). This self‐report questionnaire is made up of 19 items that reflect the spectrum of suicidal concerns: the extent of suicidal thoughts and their characteristics, as well as the patient's attitude towards them; the extent of the desire to die; the desire to make a real suicide attempt and any detail about the plans; the internal bollards to an active attempt; the subjective feelings of control and / or “courage” towards an attempt. Each item is composed of three response alternatives classified in intensity from 0 to 2. The scores of each item obtaining a total score ranging from 0 to 38. BSI has demonstrated good reliability (Cronbach's alpha coefficient equal to 0.94).

Levels of negative affectivity have been measured through the *Personality Inventory for DSM‐5‐Brief Form* (PID‐5‐BF), a 25 item self‐report inventory designed to assess the personality traits of the alternative model for personality disorders based on personality dysfunction and pathological personality traits introduced with publication of the fifth edition of the *Manual diagnostic and statistical manual of mental disorders* (DSM‐5). The tool consists of five broad domains of higher‐order traits (i.e., negative affectivity, detachment, psychoticism, antagonism and disinhibition) that correspond to five specific scales. To evaluate each item it is necessary to answer on a four‐point Likert type scale ranging from 0 (always or often false) to 3 (always or often true). In our study, the tool showed good internal consistency with Cronbach’s alpha scores ranging from 0.91 to 95 for the different domains.

### Statistical analysis

For data analysis, SPSS 25.0 software was used. Data analysis was carried out on the entire sample through the calculation of means, standard deviations and frequencies of the scores obtained by the subjects. To analyze the relationships between narcissism, emotion dysregulation and suicide ideation (both for total scales and subscales) a matrix was calculated for Pearson correlation. To accurately evaluate the predictive role of narcissism and emotion dysregulation on suicide ideation controlling for the effect of age, gender and Negative Affectivity, a multiple hierarchical linear regression was performed. In order to test the mediation hypothesis, SPSS Macro PROCESS (Hayes, 2012) was used, which allowed to verify the mediation effects, both direct and indirect, of the coefficients analyzed. The Bootstrap technique was used to calculate indirect effects.

## Results

### Differences between groups

We explored whether the two groups differed in pathological narcissism using a multivariate analysis of covariance (MANCOVA). Before carrying out the analysis, we tested the homogeneity of the variances through Levene’s test for both Grandiosity (*F*(1, 204) = 0.169, *p* = 0.681, η^2^_p_ = 0.004) and Vulnerability (*F*(1, 204) = 0.380, *p* = 0.538, η^2^_p_ = 0.143), which proved to be non‐significant for the variables in question and allowed us to carry out the comparison between groups. Results, as shown in Table 1, indicated that the two groups were significantly different on all the dimensions concerning narcissism.

**Table 1:** Means, standard deviations, and MANCOVA for pathological narcissism

	Control Group *n* = 154	Clinical Group *n* = 70		
	*M (SD)*	*M (SD)*	*F*	*p*
PNI contingent self‐esteem	2.72 (0.986)	3.63 (1.07)	**30.03**	<0.001
PNI exploitative	3.23 (0.866)	3.07 (1.02)	**7.18**	<0.001
PNI hiding the self	3.60 (0.971)	4.00 (0.837)	**8.00**	<0.001
PNI self enhancement	3.67 (0.896)	4.13 (0.924)	**15.48**	<0.001
PNI grandiose fantasy	2.64 (0.879)	2.72 (0.910)	**17.45**	<0.001
PNI devaluing	2.59 (0.979)	3.24 (0.938)	**22.04**	<0.001
PNI entitlement	3.19 (1.02)	3.22 (1.18)	**16.34**	<0.001
PNI grandiosity	3.36 (0.792)	3.47 (0.739)	**22.41**	<0.001
PNI vulnerability	2.92 (0.837)	3.66 (0.830)	**39.49**	<0.001

Note

PNI = Pathological Narcissism Inventory.

### Associations between variables

In accordance with the assumptions made, the analyses, as shown in Table 2, identified a significant positive correlation between suicide ideation score and emotion dysregulation total score (*r* = 0.324; *p* < 0.001); specifically, suicide ideation shows significant positive correlations with all emotion dysregulation subscales, except for Awareness (*p *= 0.125). The analyses also showed a significant positive correlation between suicide ideation and narcissistic vulnerability (*r* = 0.227; *p* < 0.001) and a non‐significant correlation with narcissistic grandiosity (*r* = −0.067; *p* = 0.416). Moreover, suicide ideation showed significant positive correlations with narcissism dimensions, such as Hiding the self and Devaluing (see Table 2).

**Table 2:** Partial correlation between suicidal ideation, emotion dysregulation and pathological narcissism, controlling for gender, age and negative affectivity

	1	2	3	4	5	6	7	8	9	10	11	12	13	14	15	16	17
1. BSI tot	–																
2. DERS non acceptance	0.232**	–															
3. DERS goals	0.220*	0.356**	–														
4. DERS impulse	0.213*	0.329**	0.484**	–													
5. DERS awareness	0.127	0.084	0.201*	0.212*	–												
6. DERS strategies	0.308**	0.503**	0.565**	0.672**	0.216*	–											
7. DERS clarity	0.188*	0.272**	0.294**	0.292**	0.336**	0.359**	–										
8. DERS total	0.324**	0.655**	0.714**	0.758**	0.462**	0.863**	0.571**	–									
9. PNI contingent self–esteem	0.163*	0.174*	0.328**	0.222*	0.053	0.407**	0.202*	0.354**	–								
10. PNI exploitative	−0.054	−0.033	−0.091	0.057	0.029	−0.075	−0.001	−0.034	−0.065	–							
11. PNI self enhancement	0.055	0.046	0.061	−0.08	−0.154*	0.013	−0.088	−0.038	0.298**	0.089	–						
12. PNI hiding the self	0.202*	0.171*	0.227*	0.209*	0.069	0.262**	0.244**	0.290**	0.205*	0.178*	0.192*	–					
13. PNI grandiose fantasy	−0.002	0.017	0.06	−0.066	−0.064	0.027	0.02	0.001	0.449**	0.233**	0.358**	0.261**	–				
14. PNI devaluing	0.163*	0.257**	0.179*	0.144*	−0.071	0.303**	0.152*	0.255**	0.494**	−0.022	0.126	0.305**	0.177*	–			
15. PNI entitlement	−0.156	−0.02	0.021	0.163*	−0.128	0.135	−0.187*	0.025	0.408**	0.266**	0.326**	0.114	0.486**	0.268**	–		
16. PNI grandiosity	−0.067	0.003	0.028	0.036	−0.122	0.056	−0.102	−0.009	0.437**	0.501**	0.616**	0.262**	0.792**	0.224**	0.810**	–	
17. PNI vulnerability	0.227**	0.256**	.344**	0.260**	0.032	0.449**	0.262**	0.409**	0.865**	0.013	0.296**	0.577**	0.431**	0.758**	0.387**	0.440**	–

Notes

BSI = Beck Scale for Suicidal Ideation; DERS = Difficulties in Emotion Regulation Scale; PNI = Pathological Narcissism Inventory.

BSI = Beck Scale for Suicidal Ideation Total Score; DERS = Difficulties in Emotion Regulation Scale Total Score; PNI = Pathological Narcissism Inventory Vulnerability Scale.

* *p* < 0.05; ** *p* < 0.00.

### Mediation hypotheses

We aimed to contemporary test the hypotheses that vulnerable narcissism and emotion dysregulation predict in a direct way the severity of suicide ideation and that levels of emotion dysregulation mediate the relationship between vulnerable narcissism and suicide ideation, controlling for age, gender and negative affectivity. As such, we performed this combined analysis using the macro PROCESS for SPSS (Hayes, 2012). Before testing the model, we ensured that basic assumptions for testing a mediational analysis were met, following the recommendation of Baron and Kenny (1986). So, we verified that vulnerable narcissism (i.e., PNI Vulnerability scale) was a significant predictor of BSI scores (Step 1); that vulnerable narcissism significantly predicted our mediator (i.e., DERS total score) (Step 2) and that DERS total score significantly predicted BSI scores beyond the effect of vulnerable narcissism (Step 3). Finally, we tested the mediation hypothesis (Step 4). As fully illustrated in Table 3 and in Fig, 1, we found that DERS Total score totally mediated the relationship between Vulnerable Narcissism and BSI scores.

**Table 3:** Direct and indirect effects of vulnerable narcissism on suicide ideation through emotion dysregulation

		β	SE	Bootstrap Confidence Interval [95%]
**STEP 1** PNI → BSI *R^2^* *=0*.*1871 p* = 0.00	Constant	−6.5625	3.6517	[−13.7704 to 0.6455]
	Age	0.0314	0.0535	[−0.0743 to 0.1371]
	Gender	−0.4345	1.2795	[−0743 to 0.1371]
	Negative affectivity	−2.0065	2.2811	[−6.5091 to 2.4961]
	PNI vulnerability	3.4989	0.6823	[2.1534 to 4.8443]
**STEP 2** PNI → DERS *R^2^* = 0.5509 *p* = 0.00	Constant	22.7901	6.4464	[10.0663 to 35.5138]
	Age	0.1565	0.0972	[−0.0352 to 0.3483]
	Gender	−1.7904	2.3350	[−6.3991 to 2.8184]
	Negative affectivity	20.0179	3.8821	[12.3554 to 27.6804]
	PNI vulnerability	11.6599	1.8139	[8.0796 to 15.2402]
**STEP 3** PNI + DERS → BSI *R^2^* = 0.1871 *p* = 0.00	Constant	−6.5625	3.6517	[−13.7704 to 0.6455]
	Age	0.0314	0.0535	[−0.0743 to 0.1371]
	Gender	−0.4345	1.2795	[−2.9601 to 2.0911]
	Negative affectivity	−2.0065	2.2811	[−6.5091 to 2.4961]
	PNI vulnerability	1.4368	1.1045	[−0.7433 to 3.6169]
	DERS total	0.1557	0.0416	[0.0736 to 0.2378]
**STEP 4**				
PNI → BSI		1.4368	1.1045	[−0.7433 to 3.6169]
PNI → DERS → BSI		1.8149	0.6344	[0.5200 to 3.0466]

Notes

*PNI* = Pathological Narcissism Inventory; *DERS* = Difficulties in Emotion Regulation Scale; *BSI* = Beck Scale for Suicidal Ideation.

**Fig. 1:**
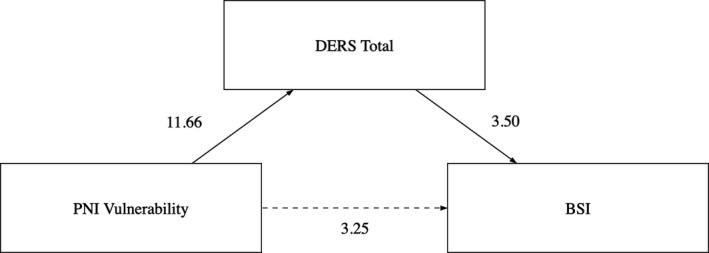
The moderation model tested.

## DISCUSSION

The present research contributes to the suicide risk investigation landscape. This study aims to better define the interplay between narcissism and emotion dysregulation in predicting suicide ideation. Not surprisingly, we found that individuals in the clinical sample showed higher levels of pathological narcissism scores. Moreover, all variables appeared to be associated with each other; in particular, suicide ideation showed significant positive correlations with all the dimensions of emotion dysregulation and with narcissistic vulnerability. These results endorse the need to consider the variables examined when exploring the specificities of the suicidal phenomenon.

Specifically, in line with the evidences that suggest the existence of different paths concerning the two dimensions of pathological narcissism (Ellison *et al*., 2013; Jauk & Kaufman, 2018) results showed both the absence of a significant association between suicide ideation and grandiose narcissism, and an association between vulnerable narcissism and suicide ideation. Specifically, the dimensions of Hiding the self and Devaluing appeared to be associated with the severity of the suicide ideation. This is in line with previous literature (see Jaksic *et al*., 2017), showing that following a narcissistic wound, narcissistic vulnerability can raise shame and mobilize narcissistic anger and intolerable anguish which can result in ideas of death.

Looking at emotion dysregulation our findings, in line with what has been highlighted by previous research (see Rajappa *et al*., 2012), confirmed that the dimension lack of awareness of emotional experiences and responses does not intervene in the development of suicidal thoughts. Moreover, data corroborate the strong association between the strategies dimension and suicide ideation, in line with the hypothesis that the feeling of helplessness in managing one’s own emotional responses, as a result of the perception of the lack of effective strategies, can contribute to suicide ideation.

Overall, considering the complex interplay between the variables investigated in the study, the most interesting finding concerns the mediating role of emotion dysregulation in the relationship between vulnerable narcissism and suicide ideation. Our data suggest that the influence of narcissistic vulnerability on suicide ideation may be affected by the difficulty in regulating negative emotions more than by negative emotions per se, which is a feature often found in pathological narcissism. In fact, it was found that emotion dysregulation totally mediates this relationship, while controlling for negative affect. This implies that an individual with narcissistic pathology who experiences negative emotions may have suicidal thoughts due to the inability to understand and regulate his affective states, rather than to the pervasive presence of negative emotions. Thus, emotion dysregulation, when combined with other risk factors (e.g., hopelessness, depression) can be turned against the individual, leading to suicide attempts (Ronningstam *et al*., 2008).

Another possible explanation to the tendency to self‐directed aggression under the influence of negative emotional states (i.e., shame) could concern the inability to go beyond what is directly perceived and to understand what others have in their minds, namely mentalization (Velotti *et al*., 2016, 2019). In narcissistic individuals, the capacity for metacognitive control, mentalization and recognition of one’s (and others’) intentions is not adequately developed, leading both to the attempt to maintain an illusory control over experiences of humiliation and shame through suicidal thoughts and to an extreme denial of the irreversibility of death, preventing the person from reflecting on the consequences of self‐destructive aggressive actions (Gabbard, 2014). Furthermore, particular aspects of emotion regulation difficulties, such as the use of other maladaptive emotion regulation strategies (e.g., rumination, the tendency to respond to a negative mood by focusing on its causes, meanings, and consequences) might be involved.

The contribution of emotion dysregulation, considering both impairments in emotion regulation and emotion regulation strategies, is an open question in understanding the mechanisms that link pathological personality traits and suicidal behaviors. This study has the merit of extending previous findings concerning community population to a clinical sample, contributing to fill the research concerning the samples to which the therapeutic interventions are addressed.

Nonetheless, some important limitations have to be discussed. The limited clinical sample size and the snowball technique adopted for recruiting control sample, does not allow for more complex statistical analyses and at the same time makes it very delicate to generalize the results obtained. Furthermore, to test the mediating role of emotion dysregulation in the relationship between narcissism and suicide ideation, we chose to concentrate our analyses only on DERS total score. A more nuanced approach could help to clarify whether some facets of emotion dysregulation have a stronger voice than others in such association.

Taken together, our findings confirm the importance of considering multiple interacting factors in the explanation of suicidal behavior. We aim to shed light on some of them, proving that personality features, such as vulnerable narcissism, play a meaningful part in suicide ideation and that its contribution is totally mediated by emotion dysregulation. As we await research that will disclose the role of single components of emotion regulation or additional intervening elements, for example, attachment representations (Pace *et al*., 2020), mentalizing abilities, alexithymia or emotion regulation strategies (Pace, Di Folco & Guerriero, 2018), our results suggest that emotion dysregulation represents one of the variables to consider when assessing suicidal risk and planning treatment for its reduction, particularly in the presence of narcissistic features.

## Conflicts of interest

The author(s) declared no potential conflict of interest with respect to the research, authorship, and/or publication of this article. The author(s) received no financial support for the research, authorship, and/or publication of this article.
